# From Small Metal Clusters to Molecular Nanoarchitectures with a Core–Shell Structure: The Synthesis, Redox Fingerprint, Theoretical Analysis, and Solid-State Structure of [Co_38_As_12_(CO)_50_]^4–^

**DOI:** 10.1021/acs.inorgchem.2c00506

**Published:** 2022-06-22

**Authors:** Roberto Della Pergola, Luigi Garlaschelli, Piero Macchi, Irene Facchinetti, Riccardo Ruffo, Stefano Racioppi, Angelo Sironi

**Affiliations:** † Dipartimento di Scienze dell’Ambiente e della Terra, University of Milano-Bicocca, Piazza della Scienza 1, 20126 Milano, Italy; ‡ Dipartimento di Chimica, University of Milano, via Venezian 21, 20133 Milano, Italy; § Department of Chemistry, Materials and Chemical Engineering, Politecnico di Milano, via Mancinelli 7, 20131 Milano, Italy; ∥ Dipartimento di Scienze dei Materiali, University of Milano-Bicocca, via Cozzi 5, 20126 Milano, Italy; ⊥ Department of Chemistry, State University of New York at Buffalo, Buffalo, New York 14260-3000, United states

## Abstract

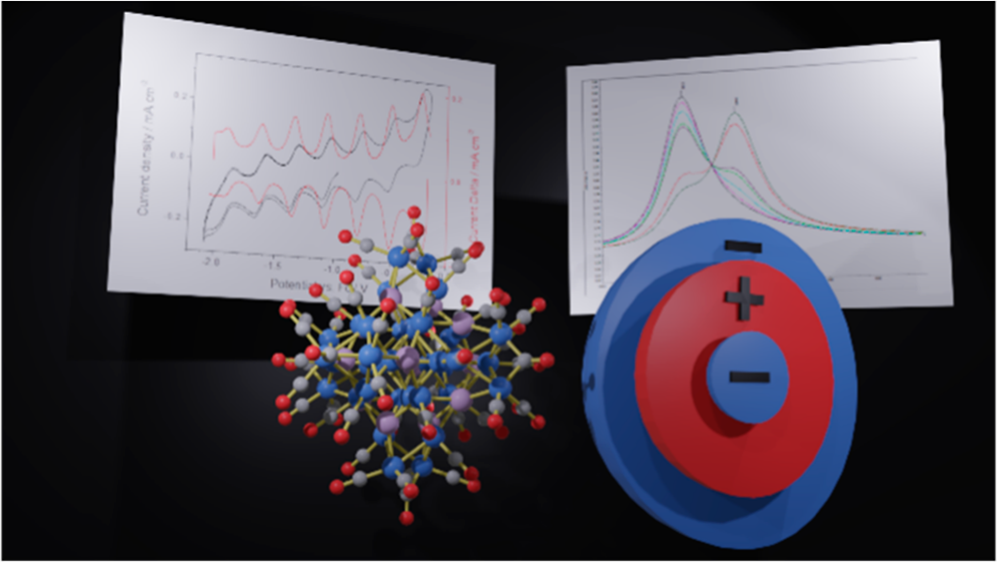

The cluster [Co_38_As_12_(CO)_50_]^4–^ was obtained by pyrolysis of [Co_6_As(CO)_16_]^−^. The metal cage features a closed-packed core inside a Co/As shell that progressively deforms from a cubic face-centered symmetry. The redox and acid–base reactivities were determined by cyclic voltammetry and spectrophotometric titrations. The calculated electron density revealed the shell-constrained distribution of the atomic charges, induced by the presence of arsenic.

## Abstract 2

The anion [Co_38_As_12_(CO)_50_]^4−^ is an ultrasmall nanometric molecular cluster. Computational analysis established that the frontier orbitals are closely spaced and that the charge is unevenly distributed on the metallic shells, as shown in the scheme. Electrochemical and spectroscopic analyses show that it reaches acid−base and redox reactivity.

## Introduction

The design of molecular architectures with different structural elements is relevant for synthetic purposes since the formation of strong bonds between metal and main group atoms may confer extra stability.^1^ Additionally, this class of compounds has been used as a single source for binary and ternary phases which,^2^ in turn, can find application as semiconductors^3^ or magnetic nanoparticles (NPs)^4^ for the deposition of thin films,^5^ for catalysis, and for electrocatalysis.^6^ In the field of semiconductors, core–shell structures have attracted considerable interest since the outer shell can modify the properties of the inner one, extending the spectrum of applications.^7^ In catalysis, elements of group 5 are particularly relevant since their presence allows good catalytic performances even under harsh conditions when the (more expensive) precious metals would be poisoned.^8^ In the last few years, cobalt phosphides have emerged as very active materials for electrocatalysis and photoelectrocatalysis, both for anodic and cathodic reactions.^9^ More recently, despite the high toxicity of As compounds,^10^ arsenides have been tested for these purposes.^11^ Thus, cluster compounds containing cobalt and an element of group 5 can find applications in these fields. Several cobalt clusters, containing one or two P atoms, either exposed or fully interstitial, have been obtained in the past and fully characterized.^12^ Instead, only a few cobalt clusters, containing heavier elements (As, Sb,^13^ and Bi^14^), are known, and their chemistry appears rich of compositional, structural, and theoretical implications. In particular, As, which can form cluster compounds, can be associated with cobalt in many different combinations. For example, the complete series As_4–*n*_{Co(CO)_3_}_*n*_ (*n* = 0,^15^ 1,^16^ 2,^17^ 3,^18^ 4^19^), where Co(CO)_3_ fragments and As atoms have interchangeable roles, was used to develop the isolobal principle.^20^ A short description of this variety can be found in reference books of inorganic chemistry.^21^ As a prosecution of our research in this field, we devoted our efforts to synthesize new Co–As clusters, trying to incorporate As atoms into small simple molecular compounds, to be employed as precursors of larger clusters and, eventually, Co–As binary phases. The cluster anion [Co_6_As(CO)_16_]^−^ was considered a suitable reagent for these investigations since it can be prepared in large amounts by a simple reaction.^22^ The cluster is formed by four edge-fused triangles, partially wrapping the As atom. The determination of its accurate solid-state structure allowed an experimental and theoretical analysis of its electron density.^23^

## Results and Discussion

Heating the potassium salt of [Co_6_As(CO)_16_]^−^ at the temperature of refluxing acetonitrile induced a fast reaction with formation of a new product, together with large amounts of [Co(CO)_4_]^−^ carbonylmetalate. The identity of the carbonyl products at this stage was confirmed by infrared (IR) spectroscopy. The IR spectrum of the product only shows two strong broad bands, corresponding to terminal and bridging ligands, in agreement with the solid-state structure (vide infra) and the density functional theory (DFT)-computed spectrum (Figure S2 in the Supporting Information). The new product was isolated in the solid state as a salt of different organic cations. We report here the crystal and molecular structure of (AsPh_4_)_4_[Co_38_As_12_(CO)_50_]·C_4_H_8_O, which gave better quality crystals. The presumable stoichiometry of the reaction is thus
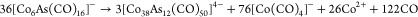
1

Even if the equation is quite cumbersome and may appear arbitrary, it accounts for the very different Co/As/charge ratio of the reactant (6:1: −1) and the product (38:12:–4), which implies that almost one-half of cobalt leaves the cluster to form mononuclear Co complexes. If the same reaction is carried out at the refluxing temperature of other high-boiling solvents (such as *n*-butanol, methylisobutylketone, and diglyme), very similar IR spectra are obtained, suggesting that the same carbonyl anions are formed. Table S1 lists the IR spectra of the reaction mixtures in these solvents.

Poor reproducibility of the IR spectra suggests that the reaction product is a mixture of [HCo_38_As_12_(CO)_50_]^3–^ and [Co_38_As_12_(CO)_50_]^4–^ anions. The deprotonation reaction is incomplete in MeCN since both species coexist in this solvent (Figure S1).
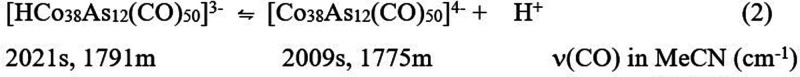
2The detection of hydride ligands in large metal clusters is very problematic, and frequently, their presence must be inferred on the basis of indirect evidence.^12c,24^ Typically, the shift in the IR bands is considered sufficient experimental evidence for the presence of H ligands in carbonyl complexes^25^ since stretching frequencies of CO are very sensitive to the amount of π*-backdonation and indirectly to the electron density of the metals. In a few cases, when the hydrides are spectroscopically or structurally detected, their different numbers strictly correspond to the variations in the IR frequencies.^26^

**Figure 1:**
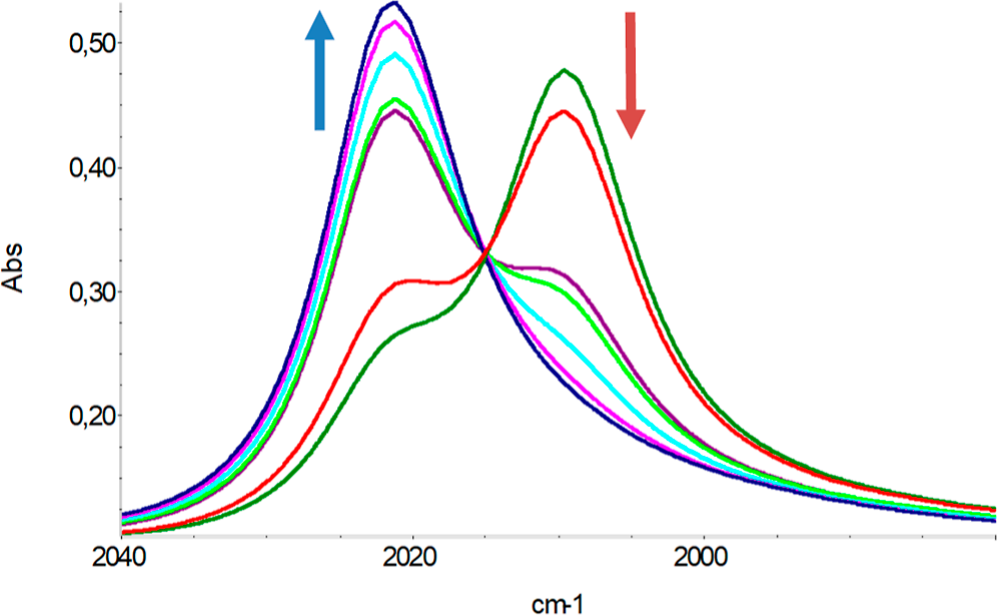
IR modifications upon adding diluted H_2_SO_4_ to a MeCN solution of [Co_38_As_12_(CO)_50_]^4–^.

In the present case, the direct detection by ^1^H NMR is hampered by the quadrupolar ^59^Co nuclei (*s* = 7/2; natural abundance, 100%). Therefore, to support our hypothesis, we performed spectrophotometric titrations of [Co_38_As_12_(CO)_50_]^4–^ with a diluted solution of H_2_SO_4_ in MeCN. The IR spectra showed band shifts at higher frequencies, with a neat isosbestic point (Figure 1) corresponding to the reverse of the above equilibrium (2).

**Figure 2:**
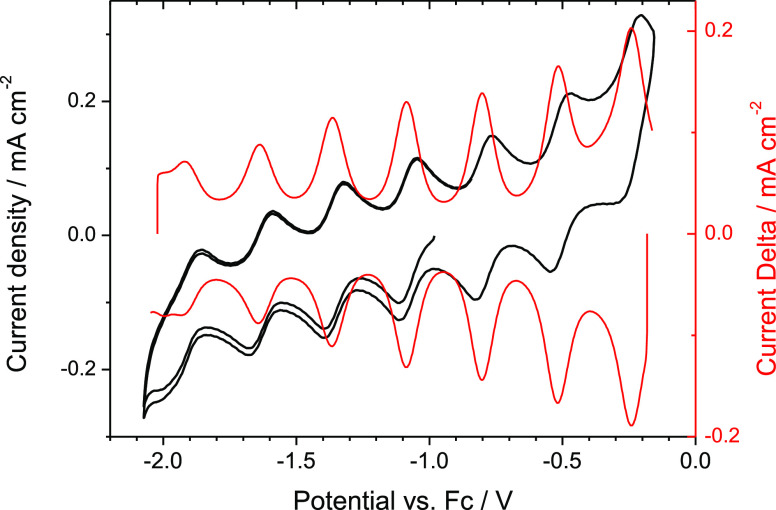
CV (black) and DPV (red) responses of 0.6 mM [Co_38_As_12_(CO)_50_]^4–^ in MeCN.

Moreover, we examined the redox aptitude of the [Co_38_As_12_(CO)_50_]^4–^ anion by cyclic voltammetry (CV) and differential pulse voltammetry (DPV) (Figure 2).

The CV profile shows, within the explored range of potentials, seven sequential electron transfer steps. Four reversible voltammetric waves are observed at potentials more negative than the open-circuit value with three more reversible waves situated at higher potentials.^27^ In total, seven redox couples are involved. Four of them, at a potential lower than −0.9 V versus Fc/Fc^+^, involve anions with a more negative charge, and the three remaining, in the anodic direction, are indicative of anions with a less negative charge. Five of them are fully reversible one-electron transfer processes while two are quasi-reversible. In particular, the last process, occurring at a very negative potential, is partially hidden by the irreversible reduction of the [PPh_4_]^+^ counterions.^28^ However, the presence of a clearly discernible anodic peak in the CV and DPV profiles supports our attribution. Table 1 lists the reversible potentials of each wave (*E*_rev_ in V) determined from DPV curves and the peak-to-peak separations (Δ*E*_p_ in mV) determined from the CV curves. Δ*E*_p_ is very close to the theoretical value of 59 mV for monoelectronic electron transfer, whereas oxidation and reduction peaks in DPV deviate less than 5 mV. Thus, this pattern shows the large redox flexibility of the cluster, which is stable in several oxidation states, accommodating all the negative charges in the range from −8 to −1 without any subsequent chemical complication.

**Table 1:** Redox Potentials and the Peak-to-Peak Separations, Listed from the Highest to the Lowest Potential

charge	–1/–2	–2/–3	–3/–4	–4/–5	–5/–6	–6/–7	–7/–8
*E*_rev_ (V vs Fc)	–0.245^a^	–0.520	–0.800	–1.085	–1.365	–1.640	–1.920^a^
Δ*E*_p_ (mV)	undetermined	63	55	71	70	90	undetermined

a Quasi-reversible waves.

The results are quite informative when compared with those of other large metal clusters. First, these redox potentials are regularly spaced, and the Δ*E*_rev_ difference between two consecutive waves is constantly 280 mV. This behavior strongly resembles that observed for large nickel carbides, which also display several reversible redox waves.^29^

The Δ*E*_rev_ gap between consecutive waves is inversely related to the number of metal atoms in the cluster core and may be indicative of the degree of metallization occurring in these atomically precise metal NPs.^30^ The Δ*E*_rev_ gap found for the [Co_38_As_12_(CO)_50_]^*n*−^ series (*n* = 1–8) is remarkably small, suggesting that the frontier orbitals of the cluster are closely spaced. The UV–vis spectrum of the anion, in MeCN solution, supports this conclusion since it shows a strong metal-to-ligand charge transfer band at high frequency and continuous featureless absorptions at higher wavelengths (Figure S3). As a matter of fact, even for small carbonyl clusters, UV–vis spectra are rarely used for characterization,^31^ whereas small maxima in the electronic spectrum of gold NPs may be associated with incipient plasmonic resonance.^32^

**Figure 3:**
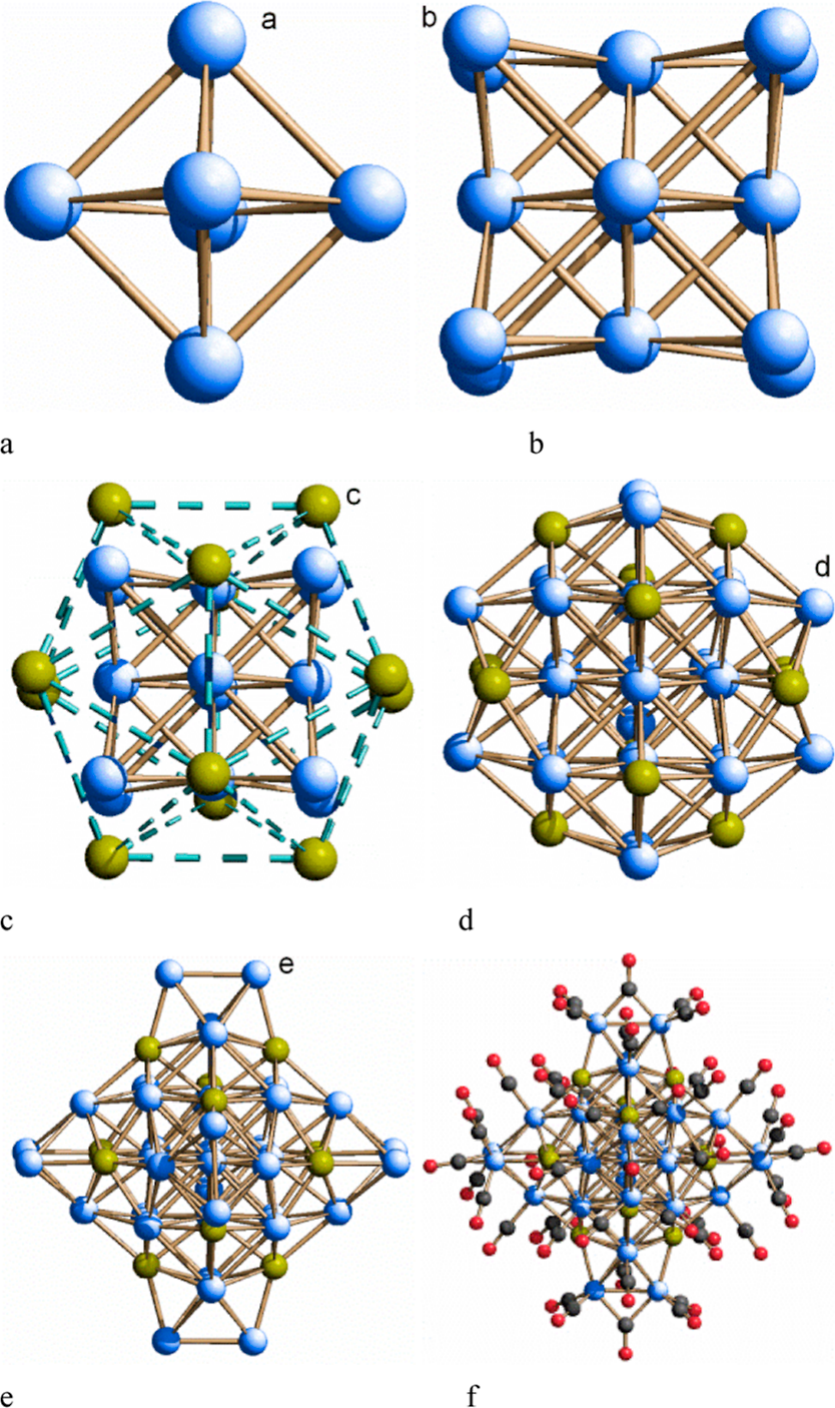
Structural units of [Co_38_As_12_(CO)_50_]^4–^. The labels are used to denote the different breakdowns of the cluster into polyhedra, as described in the text, and the different atoms which compose the same polyhedra: the internal octahedron (a) is inscribed in a cube (b), surrounded by an icosahedron of As (c) and an icosahedron of Co (d). Six capping Co_2_ units (e) complete the metal cage. The full cluster, including the carbonyl ligands, is plotted in (f). Color code: Co, blue; As, yellow; C, black; O, red.

**Scheme 1:**
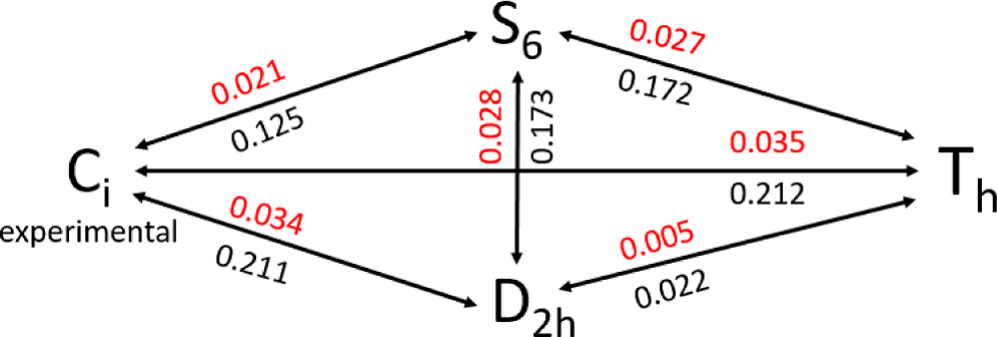
Symmetry Tree of the Geometries Discussed in the Text Reporting the rmsd (Å) among Couples of Co–As Cages (Red) and Couples of Clusters (Black) of Different Symmetries

The structure of (AsPh_4_)_4_[Co_38_As_12_(CO)_50_]·C_4_H_8_O was determined by X-ray diffraction on single crystals of the species. In the triclinic *P*1̅ space group, anions and cations are packed in a 1:4 ratio, with the anion lying on the crystallographic inversion center. Owing to the very soft nature of the large monovalent (AsPh_4_)^+^ cations, lacking strongly interacting functional groups, as well as the cocrystallized tetrahydrofuran (THF) solvent, the anion geometry is poorly affected by the crystal field. The [Co_38_As_12_(CO)_50_]^4–^ anion possesses a truncated octahedral cage (Co_26_As_12_, Figure 3d), with all exposed Co atoms bound to a terminal CO ligand [Co_26_As_12_(CO)_20_], capped by six dangling Co_2_(CO)_5_ dimers [Co_38_As_12_(CO)_50_, Figure 3f]. This unprecedented geometry can be rationalized on the basis of its *T*_h_ pseudo-symmetry [root mean square deviation (rmsd) from the experimental geometry: cage, 0.035 Å; cluster, 0.21 Å], although the only crystallographic operator is the inversion center and the closest high-symmetry group is S_6_ (rmsd: cage, 0.021 Å; cluster, 0.12 Å) (Scheme 1).

Under this assumption, the 12 As atoms are all pseudo-equivalent, while the 38 Co atoms are grouped in four classes of pseudo-equivalent atoms. As shown in Figure 3, the metal cage can be easily described by a shell structure where an octahedral core of Co [Co(a), distance from the cluster center (*r*) = 1.75 Å, **3a**] is surrounded by a cube of Co [Co(b), *r* = 3.30 Å, **3b**] contained in a truncated octahedron [resulting from two interpenetrated icosahedra of 12 As (*r* = 3.72 Å, **3c**) and 12 Co(d) (*r* = 4.09 Å, **3d**) atoms, respectively] whose six “square faces” support six dangling Co(e)–Co(e) edges (parallel to the As···As diagonals) which define the fourth (partial) shell (*d* = 5.60 Å, **3e**). Due to the irregularity of the two icosahedra (particularly the cobalt one, the rmsd from the idealized *I*_h_ symmetry is 0.68 Å), the truncated octahedron has concave “square faces” actually resembling butterfly cavities [with markedly different Co(d)···Co(d) and As···As diagonals, 3.11 and 3.78 Å, respectively].

**Figure 4:**
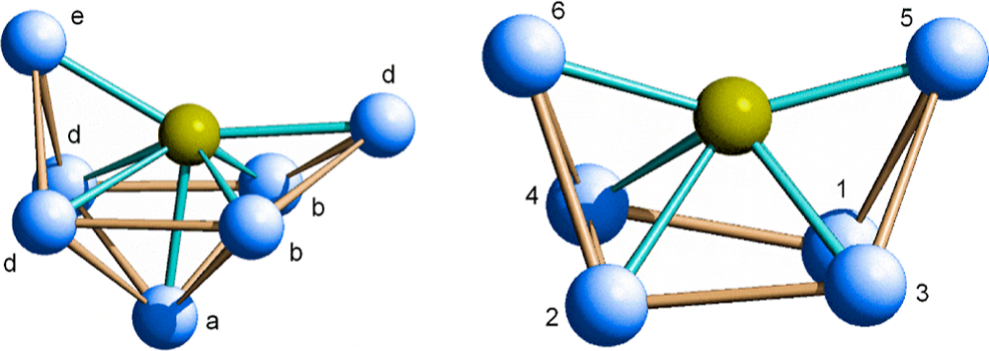
Inverse coordination^37^ of As in [Co_38_As(CO)_50_]^4–^ (left) and in [Co_6_As(CO)_16_]^−^ (right). The Co atoms on the left are labeled as in Figure 3 and those on the right are numbered according to ref (23). Color code: Co, blue; As, yellow.

The cluster is completed by 50 carbonyl ligands (**3f**) bound to all Co atoms but the inner ones. The particle thus has a diameter of ca. 16 Å. Noteworthy, the main distortions from the *T*_h_ symmetry (leading to S_6_, rmsd 0.12 Å) arise from the configurational freedom of the Co(e)–Co(e) edges and their CO ligands. The presence of such dangling units is due to the stereoelectronic requirements of the As atoms allowing the formation of Co_7_As envelops related to the Co_6_As one of the precursor [Co_6_As(CO)_16_]^−^.^22^ Noteworthily, the presence of a seventh coordinated metal [namely, Co(a)] enlarges the capped “square face” and reduces the “exposition” of the As atom [the Co(e)–As–Co(d) angle is 146 vs 150°] (Figure 4).

The whole structure shows the transition from a closely packed inner core of a pure metallic composition to the less regular shape of the outer Co_12_As_12_ shell. Noteworthily, the average Co–Co distance in the core is even smaller than in that in metallic Co: d_Co–Co_ is 2.468(2) Å in the inner shell, whereas it is 2.505 Å in the hexagonal closed packed phase of Co (stable under ambient conditions) and 2.513 Å in the high-temperature cubic closed packed structure. As we will discuss below, the metallic core is quite electron withdrawing, and this implies that more electrons are available to stabilize the Co_6_ inner unit, which therefore shrinks.

The cluster shell structure can be therefore related to that of core–shell quantum dots, formed by layers of different composition,^33^ or, more loosely, to thiolate-protected NPs, where a fully metallic icosahedral core is surrounded by an outer layer of staple arrays where additional Au atoms are held in close contact to the cluster surface by the S atoms of the ligands.^34^

The total number of cluster valence electrons (CVEs) of [Co_38_As_12_(CO)_50_]^4–^ is somewhat ambiguous because of the “semiexposed” stereochemistry of As atoms, which cannot be considered a priori as three or five electron donors (interstitial atoms of main groups usually donate all valence electrons). However, by comparing it with that of [Pt_38_(CO)_44_]^2–^ (470), a cubic closed packed (*ccp*) cluster with the same number of metal atoms, it is possible to evaluate both the degree of perturbation of the cluster “compactness” inherent to the presence of the As atoms and their formal donor properties to CVEs. Compact carbonyl clusters have largely delocalized metal–metal bonds and roughly follow the 6N + 7 cluster valence orbital (CVO) rule.^35^ The presence of a larger CVO number is normally associated to a loss of compactness and to a more localized bond description, eventually leading to the fulfillment of the effective atomic number (EAN) rule.

Given that the Co_38_As_12_ cage has a clear boundary between bonded (all <2.70 Å) and nonbonded Co/Co interactions (all >3.14 Å), the EAN foresees unambiguously 480 CVEs for a cluster of 38 metals with 102 edges (38 × 18 – 102 × 2 = 480). This well supports the hypothesis that As is a three-electron donor (38 × 9 + **12 × 3** + 50 × 2 + 4 = 482 CVEs) rather than a five-electron donor (506 CVEs). The two unpredicted “extra” electrons being reasonably related to the octahedral core (it is well known that octahedral clusters are two electrons richer than expected, 86 vs 84 CVEs).

Eventually, the comparison between the 482 CVEs of [Co_38_As_12_(CO)_50_]^4–^ (EAN: 480) and 470 CVEs of [Pt_38_(CO)_44_]^2–^ (EAN: 396) suggests that each As ligand promotes one “more” CVE by leading to a far less compact cluster, thus hampering multicenter, delocalized metal–metal bonds but in the central octahedron.

As anticipated above, [Co_38_As_12_(CO)_50_]^4–^ could be idealized in the T_h_ point symmetry group. However, the computational analysis of the molecular orbitals revealed that the frontier orbitals are threefold degenerated, and they transform as the irreducible representation *t*_u_ but host only four electrons. Consequently, the system naturally lowers its symmetry either to the subgroup D_2h_ or S_6_, through a mild first-order Jahn–Teller distortion (an rmsd of only 0.02 Å from an idealized T_h_ symmetry).

To assess how relevant it is, we optimized [Co_38_As_12_(CO)_50_]^4–^ in both the D_2h_ and S_6_ point groups using DFT calculations. The two structures differ by only 0.33 kcal/mol (in favor of S_6_), which is well within the accuracy of the calculation. Moreover, the distribution of the atomic charges is unaltered (see Table S2), confirming that D_2h_ and S_6_ are electronically very similar.

A topological analysis of the theoretical electron density on the D_2h_ cluster reveals a well-defined shell separation of the atomic charges (Figure S4). In the core of the cluster, the Co(a) internal octahedron (Figure 4) is predicted to carry a total negative charge of ca. 0.7 electrons [−0.11 for each Co(a)]. Moving toward the external shells, cobalt atoms turn positive and progressively increase the charge: from Co(b)^+0.17^ to Co(d)^+0.25^ and eventually to Co(e)^+0.40^. The As^–0.13^ atoms, lying between Co(b) and Co(d), partially neutralize the cobalt positive charges, producing an ideal Co(b)–As–Co(d) shell globally charged +2.7. Consequently, the net negative charge of the cluster is mostly concentrated in the external carbonyls and only partially in the octahedral core.

Topologically, all the Co–Co interactions are comparable in strength (judging from the electron density at the bond critical points) between each other and observable prevalently between shells, that is, Co(a)–Co(b), Co(b)–Co(d), and Co(d)–Co(e). Only the internal core presents intershell Co(a)–Co(a) interactions. On the other hand, As atoms are found to be topologically tightly connected with all the cobalt atoms, with strengths that increase with the positive charge of Co (see Table 2).

**Table 2:** Experimental and Theoretical^b^ Average Bonding Parameters (with Standard Deviations in Parenthesis)^a^

classes	*m*^d^	*r* [Å]^e^	average atomic charges	Co(a)	Co(b)	As	Co(d)	Co(e)
**Co(a)**	6	1.75	–0.11	**4**	**4**	**2**	**2**	
				2.468(2)	2.701(7)	2.392(2)	2.569(7)	
				*2.544(5)*	*2.665(3)*	*2.388(8)*	*2.555(6)*	
				***0.296(2)***		***0.486(6)***	***0.300(7)***	
				0.42(6)	0.25(1)	0.64(2)	0.30(3)	
**Co(b)**	8	3.30	0.17	**3**		**3**	**3**	
				2.701(7)		2.307(6)	2.69(2)	
				*2.665(3)*		*2.283(2)*	*2.734(9)*	
						***0.550(2)***		
				0.25(1)		0.664(4)	0.250(4)	
**As**	12	3.72	–0.13	**1**	**2**		**3**^f^	**1**
				2.392(2)	2.307(6)		2.52(2)	2.327(7)
				*2.388(8)*	*2.283(2)*		*2.55(4)*	*2.348(2)*
				***0.486(6)***	***0.550(2)***		***0.35(3)***	***0.603(2)***
				0.64(2)	0.664(4)		0.41(6)	0.656(3)
**Co(d)**	12	4.09	0.25	**1**	**2**	**3**^f^		**2**
				2.569(7)	2.69(2)	2.52(2)		2.64(3)
				*2.555(6)*	*2.734(9)*	*2.55(4)*		*2.605(3)*
				***0.300(7)***		***0.35(3)***		***0.300(1)***
				0.30(3)	0.250(4)	0.41(6)		0.444(3)
**Co(e)**	12	5.60	0.40			**1**	**2**	**1**
						2.327(7)	2.64(3)	2.58(1)
						*2.348(2)*	*2.605(3)*	*2.70(2)*
						***0.603(2)***	***0.300(1)***	
						0.656(3)	0.444(3)	0.268(6)

a In each box are reported, in order, the number of equivalent interactions between atoms of different classes (**bold**); the experimental bond distance (in Å, normal); the theoretical bond distances (in Å, *italics*); the electron density at the critical point (ρ_bcp_ [e·Å^–3^], ***bold italics***); and the delocalization index (underlined).^c^

b Experimental bond distances are averaged according to the T_h_ idealized symmetry; theoretical values are averaged according to the D_2h_ point group.

c Electron densities and delocalization indices are averaged over the entire class.

d Multiplicity of the class in the idealized T_h_ symmetry.

e Distance from the cluster center.

f Each Co(d) is bonded to three As atoms belonging to two butterflies.

Finally, from the analysis of the molecular orbitals (Table S3), it can be seen that the frontier orbitals are primarily localized on the cobalt atoms. On average, for the range from the highest occupied molecular orbital (HOMO) – 8 to the lowest unoccupied molecular orbital (LUMO) + 8, more than 60% of each molecular orbital is generated by Co(a), Co(b), and Co(d) contributions. Consequently, the reduction–oxidation cycle of [Co_38_As_12_(CO)_50_]^4–^ (see CV results) will primarily involve the storing/draining of electrons to/from the internal cobalt shells, respectively.

The condensation of several [Co_6_As(CO)_16_]^−^ into a single [Co_38_As_12_(CO)_50_]^4–^ recalls somehow the growth of nanosemiconductors, where monomeric precursors are assembled into small intermediates, and their dimensions are allowed to increase.^36^ Thanks to the similar inverse coordination^37^ of As by Co in the reactant and in the product, a comparison of the bonding features, computationally obtained for both, allows us to understand how the Co–As interactions are modified after the growth and how the atomic charges are redistributed between the two elements. The computed electron density at the critical points and the atomic charges are reported in Table 3, allowing direct comparisons. It is evident that the charge separation is reduced in [Co_38_As_12_(CO)_50_]^4–^ (As is less negative and Co less positive) and that the formation of an additional Co(a)–As bond marginally affects the Co–As bond in the basal square but reduces more deeply the electron density over the “capping” Co–As bonds.

**Table 3:** Atomic Charges and Electron Density at the Bond Critical Points Calculated for the Clusters of [Co_38_As_12_(CO)_50_]^4–^ and [Co_6_As(CO)_16_]^−^ (Which has a Pseudo-*C*_2_ Symmetry)

Co_38_As_12_			Co_6_As		
Co label	ρ(Co–As)	charge	Co label	ρ(Co–As)	charge
Co(a)	0.49	–0.11	Co(1), Co(2)	0.41	+0.43
Co(b)	0.55	+0.17	Co(3), Co(4)	0.50	+0.42
Co(d)	0.35	+0.25	Co(5), Co(6)	0.56	+0.37
Co(e)	0.30	+0.40			
Co(e,d)	0.32	+0.32	Co(5,6)	0.56	+0.37
Co(b,b,d,d)	0.45	+0.21	Co(1–4)	0.46	+0.42
As		–0.13	As		–0.22

## Conclusions

We have prepared and fully characterized the [Co_38_As_12_(CO)_50_]^4–^ cluster anion, which combines a transition metal and a main group element. Single-crystal X-ray diffraction allowed us to establish its unprecedented structure, which progressively evolves from the ccp structure of a pure metal (corresponding to the high-temperature phase of Co) to a molecular nano-sized particle (with a diameter of ca. 1.6 nm). The DFT bonding analysis shows that the “doping” of As atoms induces an uneven charge distribution: negative in the inner shell and progressively positive in the outermost metal shell, eventually compensated by the π*-acidic carbonyl ligands. These properties characterize these molecules as an atomically precise model of core–shell quantum dots. Spectrophotometric titration with dilute acids suggests that the cluster can add hydride ligands, and electrochemical investigations show that all the anionic species [Co_38_As_12_(CO)_50_]^*n*^ (*n* = −1 to −8) can be obtained by redox reactions. Further experiments are planned in order to establish whether the stoichiometric addition of electrons and protons to this cluster can be exploited for the catalytic production of hydrogen, as found for other interstitial cobalt clusters.^38^

## Experimental Section

All the solvents were purified and dried by conventional methods and stored under nitrogen. All the reactions were carried out in an oxygen-free nitrogen atmosphere using the Schlenk tube technique.^39^ IR spectra in solution were recorded on a Nicolet iS10 spectrophotometer using calcium fluoride cells previously purged with N_2_. All manipulations with arsenic(V) solution must be conducted in a properly working chemical fume hood, glovebox, or other suitable containment device. Proper personal protection equipment (safety glasses with side shields, a laboratory coat, and protective nitrile gloves) must be worn at all times. All disposable materials contaminated with arsenic must be disposed as hazardous waste. The solution used for electrochemical measurement contains perchlorate salts. It should not be dried or heated since organic solutions containing perchlorate salts are capable of violent explosions during evaporation.

A batch of Na[Co(CO)_4_] was prepared by dissolving 5 g of Co_2_(CO)_8_ in anhydrous THF (50 mL) and allowing it to react with small pieces of Na (0.8 g) until the IR bands of the reactant disappeared (2–3 days). The pale solution was filtered, and THF was dried in vacuum, under moderate heating, to remove all traces of the solvent. ν(CO) IR bands in THF: 2010 vw, 1887 vs, 1857 m cm^–1^.

### Synthetic Procedure

The K[Co_6_As(CO)_16_] salt was prepared with a slight modification in the literature method.^22^ In a typical synthetic procedure, 1.80 g of Na[Co(CO)_4_] and 0.82 g of hydrated As_2_O_5_ were allowed to react at room temperature (RT) for 72 h in 70 mL of THF. The Co/As ratio was slightly adjusted in order to minimize the amount of [Co(CO)_4_]^−^ or other undesired byproducts. The mixture was filtered, and the solvent was evaporated under vacuum. The black oily residue was purified by suspending it in 60 mL of an aqueous solution of KBr (20%). After stirring for 8 h, the pale mother liquor was removed using a syringe, and the dark microcrystalline powder was dried in vacuum. The solid, constituted by K[Co_6_As(CO)_16_], was dissolved in MeCN (50 mL) and filtered. Its identity and purity were checked by comparing its IR spectra with literature data.^22^ The solution was heated under reflux for 8 h, filtered, and evaporated to dryness.

The solid obtained at this stage is soluble in most organic solvents, such as MeOH, THF, and acetone. Its IR spectra show that it is composed by the salts of [Co_38_As_12_(CO)_50_]^4–^ and [Co(CO)_4_]^−^ anions. It can be treated differently depending on the final purposes.

In order to obtain good quality crystals, the solid was dissolved in a minimum amount of THF and layered with a solution of AsPh_4_Cl in 2-propanol (ca. 0.5 mg/mL). After diffusion was completed, the mother liquors were removed using a syringe.

The black crystals of (AsPh_4_)_4_[Co_38_As_12_(CO)_50_]·C_4_H_8_O were washed with 2-propanol, dried, and used for X-ray analysis.

Calc for C_150_H_88_As_16_Co_38_O_51_: C, 29.32; H, 1.44%; found C, 29.1; H, 1.2%.

To obtain larger amounts of the compound, to be used for chemical characterization, the same solid was dissolved in methanol. Addition of triphenylphosphonium bromide (PPh_4_Br) (or other ammonium halides) caused precipitation of the insoluble final product, which could be thus separated from most of the Co(II) and Co(-1) byproducts. After complete precipitation occurred, the solid was collected by filtration, washed with 2-propanol, and dried. The final yield was 20% (150 mg). The ammonium, phosphonium, and arsonium salts are only soluble in MeCN, DMF, and DMSO.

ν(CO) IR bands in MeCN solution: 2009s, 1775m, br. Calc for (PPh_4_)_4_[Co_38_As_12_(CO)_50_] (C_146_H_80_As_12_Co_38_O_51_P_4_): C, 29.74; H, 1.37%; found C, 29.8; H, 1.1%

### Electrochemical Measurements

Test solutions were prepared by dissolving the salt in 0.1 M tributylammonium percolate (TBAClO_4_) in acetonitrile (CH_3_CN). The concentration of electroactive species was 0.6 mM. DPV and CV were carried out at scan rates of 20 and 50 mV/s, respectively, using a PARSTA2273 potentiostat in a three-electrode electrochemical cell. The counter electrode was held in a cell arm separated by a porous glass frit. All the measurements were performed in a glovebox filled with N_2_ ([O_2_] and [H_2_O] ≤ 0.1 ppm). The working, counter, and the pseudo-reference electrodes were a glassy carbon pin, a Pt flag, and a Ag/AgCl wire, respectively. The working electrode discs were well polished with a 0.1 μm alumina suspension, sonicated for 15 min in deionized water, and washed with 2-propanol. The Ag/AgCl pseudo-reference electrode was calibrated by adding ferrocene (5 × 10^–3^ M) to the test solution before and after each CV and DPV measurement. The values of *E*° of the Fc^+^/Fc couple do not differ more than 5 mV between the measurements. In the CV measurement, the Fc^+^ wave shows a difference between the oxidation and reduction peaks of 70 mV. All the measurements were performed at RT (±5 mV).

### Crystal Structure Analysis

C_150_H_88_O_51_Co_38_As_16_, *M*_r_ = 6114.3; triclinic, space group *P*1̅ (N° 2) *a* = 16.026(5) Å, *b* = 17.956(5) Å, *c* = 19.651(5) Å; α = 110.379(5)°; β = 110.782(5)°; γ = 101.013(5)°; *V* = 4626(2) Å^3^. *D*_calc_ = 2.206 g cm^–3^, *Z* = 1. A total of 20,340 intensities were recorded at 298 K on a SMART CCD diffractometer (0 < 2θ < 50°, Mo Kα radiation, detector to sample distance = 5.4 cm, 2500 frames collected, 20 s per frame, ω-scan method). An empirical absorption correction (SADABS) was applied. The structure was solved by direct (SIR97) and refined (SHELX) methods by full-matrix least-squares (on *F*_o_^2^) on the basis of all 13,067 independent reflections with *I* > 0. Anisotropic thermal factors were assigned to all nonhydrogen atoms but to the disordered clathrate THF molecule; hydrogens were riding in the idealized position. The final values of agreement indexes *R*_1_ and *wR*_2_ were, respectively, 0.0819 [0.0384 for reflections with *I* > 2σ(I)] and 0.0764. The full table with crystallographic, collection, and refinement data is given in the Supporting Information.

### DFT Calculations

DFT calculations were performed with Gaussian09^40^ at BLYP-D3BJ/SBKJC-ECP and LC-BLYP/SBKJC-ECP, respectively, for the optimization geometry and the single point on the optimized geometry.^41^ The system was considered in the singlet spin and relaxed states in the point group D_2h_ after testing both the D_2h_ and S_6_ geometries. The YQC algorithm was used to reach the convergence in the self-consistent field (SCF) procedure,^42^ density fitting was used to solve the Coulomb problem,^43^ and “Loose” convergence criterions were imposed for the geometry optimization. The program package AIMALL^44^ was used for the topological analysis of the theoretical electron density.

## Supporting Information Available

IR spectra in MeCN of pure [Co_38_As_12_(CO)_50_]^4–^, pure [HCo_38_As_12_(CO)_50_]^3–^ and an as-prepared solution of (PPh_4_)_3_[HCo_38_As_12_(CO)_50_]; computed IR spectrum of [Co_38_As_12_(CO)_50_]^4–^ (D_2h_) in the zone of the CO stretching; UV spectrum of [Co_38_As_12_(CO)_50_]^4–^ in MeCN; schematic shell-structure representation of [Co_38_As_12_(CO)_50_]^4–^ with the separation of shell charges; IR spectra of the reaction mixtures obtained by heating [Co_6_As(CO)_16_]^−^ in other solvents; atomic Bader charges in [Co_38_As_12_(CO)_50_]^4–^ calculated from the geometries optimized in the D_2h_ and S_6_ point groups; molecular orbital energies, symmetries, and percentage of the “shell’s contribution” to the molecular orbital; crystal data and details of the structure determination; and computed structures and Cartesian coordinates for [Co_38_As_12_(CO)_50_]^4–^ in the D_2h_ and S_6_ geometries. Crystallographic data for the structural analysis have been deposited with the Cambridge Crystallographic Data Centre, CCDC reference number 2128149 (PDF)
