# Supplementary material for: From Small Metal Clusters to Molecular Nanoarchitectures with a Core–Shell Structure: The Synthesis, Redox Fingerprint, Theoretical Analysis, and Solid-State Structure of [Co38As12(CO)50]4–
Source: Inorg Chem. 2022 Jun 22;61(26):9888–96. doi: 10.1021/acs.inorgchem.2c00506 (PMC9937531; doi:10.1021/acs.inorgchem.2c00506)
Supplement: Supplementary file 1 — ic2c00506_si_001.pdf [file ic2c00506_si_001.pdf]

Supplementary Information

From Small Metal Clusters to Molecular Nanoarchitectures with Core-Shell Structure: Synthesis, Redox Fingerprint, Theoretical Analysis and Solid-State Structure of $[Co_{38}As_{12}(CO)_{50}]^{4-}$.

By

Roberto Della Pergola,[a,] Luigi Garlaschelli,[b] Piero Macchi,[c] Irene Facchinetti,[d] Riccardo Ruffo,[d] Stefano Racioppi,[b,e] and Angelo Sironi[b,*].*

a. University of Milano-Bicocca, Dipartimento di Scienze dell'Ambiente e della Terra, piazza della Scienza 1 – 20126 Milano, Italy. Email: roberto.dellapergola@unimib.it
b. University of Milano, Dipartimento di Chimica, via Venezian 21 – 20133 Milano, Italy. Email:angelo.sironi@unimi.it
c. Politecnico di Milano, Department of Chemistry, Materials and Chemical Engineering, via Mancinelli 7, 20131, Milano, Italy. Email: piero.macchi@polimi.it
d. University of Milano-Bicocca, Dipartimento di Scienze dei Materiali, via Cozzi 5 – 20126 Milano, Italy
e. State University of New York at Buffalo, Department of Chemistry, Buffalo, New York 14260-3000, United States.

This Electronic Supplementary Information contains:

Figure S1 - The IR spectra in MeCN of a) $[Co_{38}As_{12}(CO)_{50}]^{4-}$ b) an as-prepared solution of $(PPh_4)_3[HCo_{38}As_{12}(CO)_{50}]$ c) the (b-a) difference spectrum, assignable to pure $[HCo_{38}As_{12}(CO)_{50}]^{3-}$

Figure S2 - The computed IR spectrum of $[Co_{38}As_{12}(CO)_{50}]^{4-}$ (D_{2h}) in the zone of the CO stretching

Figure S3 - The UV spectrum of $[Co_{38}As_{12}(CO)_{50}]^{4-}$ in MeCN.

Figure S4 - Schematic shell-structure representation of $[Co_{38}As_{12}(CO)_{50}]^{4-}$ with the separation of shell's charges, LC-BLYP/SBKJC-ECP level of theory.

Table S1 – IR spectra of the reaction mixtures obtained by heating $[Co_6As(CO)_{16}]^-$ in other solvents

Table S2 - Atomic Bader charges in $[Co_{38}As_{12}(CO)_{50}]^{4-}$ calculated from the geometries optimized in the D_{2h} and S_6 point groups.

Table S3 - Molecular orbital energies [eV], symmetries and percentage of the "shell's contribution" to the molecular orbital, LC-BLYP/SBKJC-ECP level of theory.

Table S4 - Crystal Data and Details of the Structure Determination for: mart3 P -1 R = 0.04

Table S5 - Computed Structures and Cartesian Coordinates for $[Co_{38}As_{12}(CO)_{50}]^{4-}$ in the D_{2h} and the S_6 geometries, optimized at the BLYP-D3BJ/SBKJC-ECP level of theory.

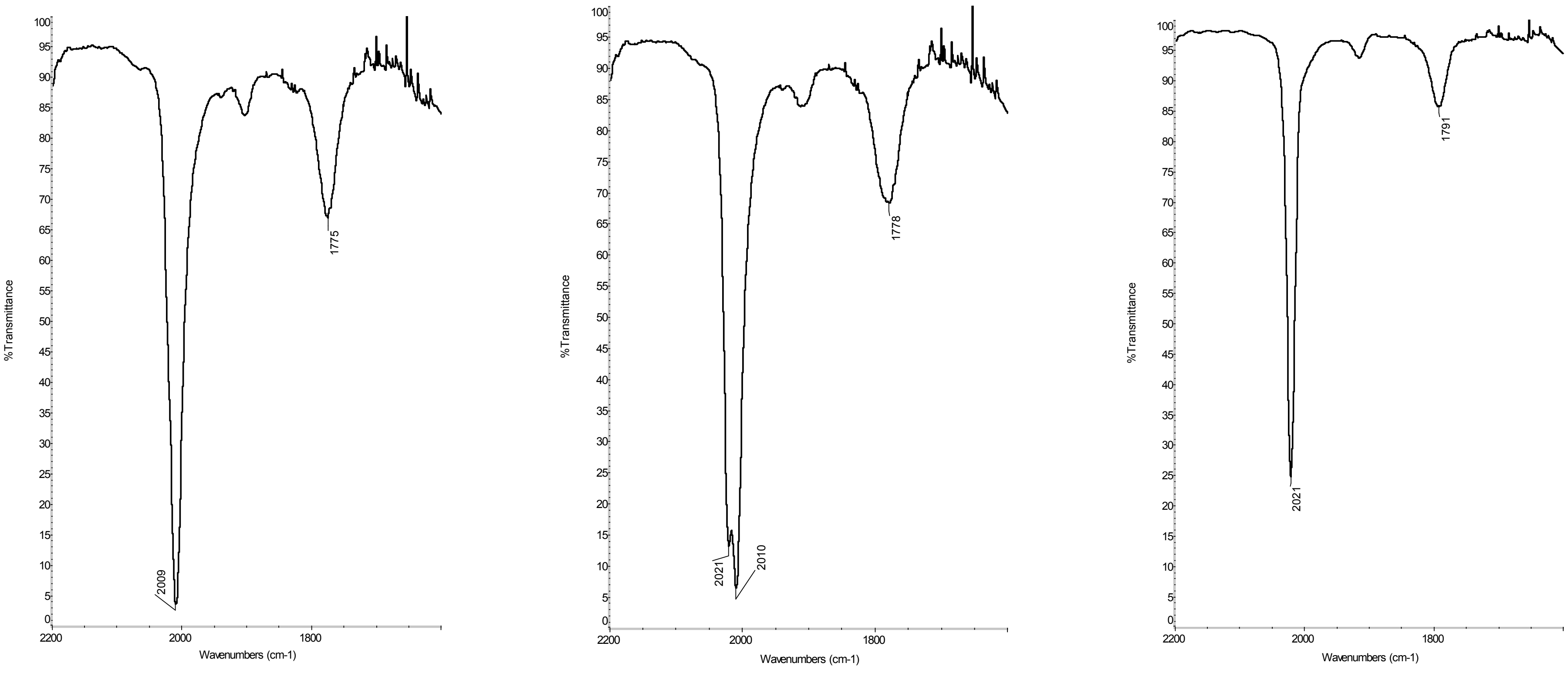

a **b** **c**

Figure S1 The IR spectra in MeCN of a) $[Co_{38}As_{12}(CO)_{50}]^{4-}$ b) an as-prepared solution of $(PPh_4)_3[HCo_{38}As_{12}(CO)_{50}]$ c) the (b-a) difference spectrum, assignable to pure $[HCo_{38}As_{12}(CO)_{50}]^{3-}$

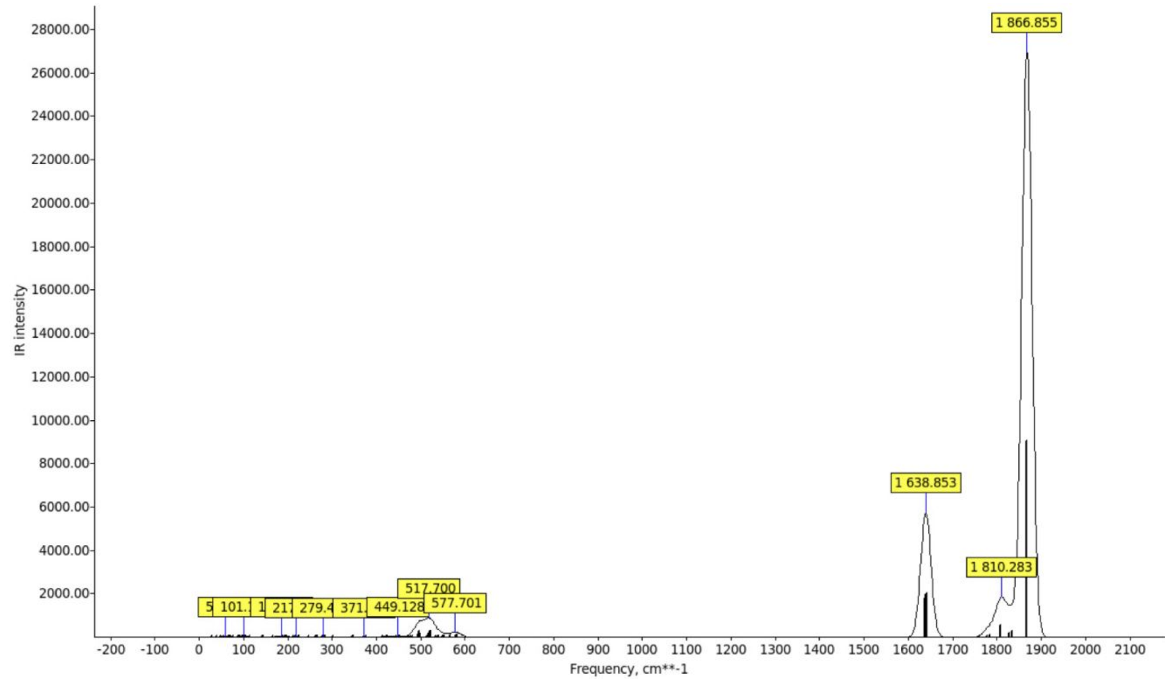

Fig S2 – The computed IR spectrum of $[Co_{38}As_{12}(CO)_{50}]^{4-}$ (D_{2h}) in the zone of the CO stretching, LC-BLYP/SBKJC-ECP level of theory.

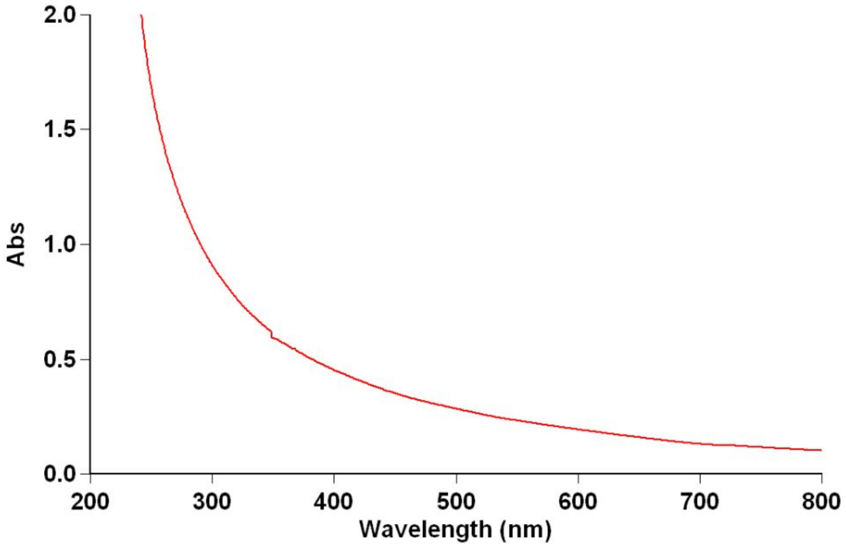

Fig S3 – The UV spectrum of $[Co_{38}As_{12}(CO)_{50}]^{4-}$ in MeCN.

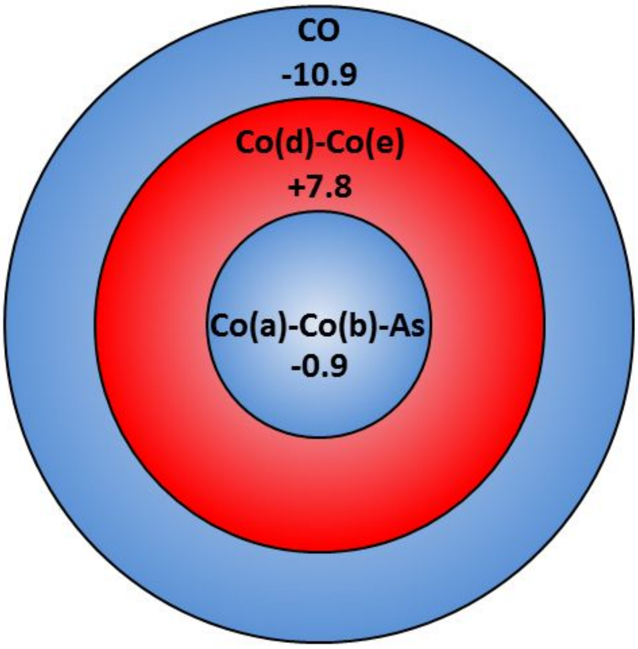

Figure S4. Schematic shell-structure representation of $[Co_{38}As_{12}(CO)_{50}]^{4-}$ with the separation of shell's charges, LC-BLYP/SBKJC-ECP level of theory.

Table S1 – IR spectra of the reaction mixtures obtained by heating $[Co_6As(CO)_{16}]^-$ in other solvents

Solvent	Boiling point (°C)	Time (h)	IR bands	Other details
THF	66	6	2001s, 1811w	
MeCN	82	8	2021s, 1892vs, 1795w	Main product identified as $[Co_{38}As_{12}(CO)_{50}]^{4-}$
1-propanol	97	8	2019sh, 2002s, 1903s, 1796m	Main product identified as $[Co_{38}As_{12}(CO)_{50}]^{4-}$
Methylisobutylketone	117	11	2020sh, 2005s, 1798w	
n-butanol	118	11	2031s, 1813m	Main product identified as $[Co_{38}As_{12}(CO)_{50}]^{4-}$
Diglyme	162	3	2019s, 1797w (MeCN)	Products insoluble in diglyme

Table S2 Atomic Bader charges in $[Co_{38}As_{12}(CO)_{50}]^{4-}$ calculated from the geometries optimized in the D_{2h} and S_6 point groups.

Level of Theory	LC-BLYP	BLYP	BLYP
Point Group	D_{2h}	D_{2h}	S_6
Co(a)	-0.11	-0.04	-0.04
Co(b)	0.17	0.23	0.23

Co(d)	0.25	0.32	0.33
Co(e)	0.40	0.56	0.56
As	-0.13	-0.16	-0.15

Table S3 Molecular orbital energies [eV], symmetries and percentage of the "shell's contribution" to the molecular orbital, LC-BLYP/SBKJC-ECP level of theory.

MO	E [eV]	Symmetry	Co(a)	Co(b)	Co(d)	Co(e)	As	Carbonyls
L+8	4.630	B1g	14	27	10	16	15	18
L+7	4.430	B1g	14	19	24	16	6	20
L+6	4.420	B2g	5	25	14	23	12	21
L+5	4.390	B3g	21	20	23	14	4	19
L+4	4.370	B2u	18	11	50	1	7	14
L+3	4.320	B2g	31	22	18	9	6	14
L+2	4.170	B1u	9	18	38	1	20	14
L+1	4.080	B3u	11	17	39	1	18	14
LUMO	3.880	B2u	14	23	26	6	19	11
HOMO	-0.150	B1u	25	16	32	9	7	12
H-1	-0.23	B3u	23	17	30	10	9	11
H-2	-0.67	Au	36	7	12	14	19	11
H-3	-0.720	B2u	17	22	14	13	20	13
H-4	-0.73	Au	32	8	14	14	20	12
H-5	-0.94	Ag	17	27	24	5	21	7
H-6	-1.03	B2g	29	21	6	15	16	12
H-7	-1.07	B3u	18	18	22	13	16	13
H-8	-1.07	B3g	24	23	6	16	20	12

Table S4 - Crystal Data and Details of the Structure Determination for: mart3 P -1 R = 0.04

Formula	C50 As12 Co38 O50, 4(C24 H20 As), C4 O
Formula Weight	6136.20
Crystal System	triclinic
Space group	P-1 (No. 2)
a, b, c [Angstrom]	16.026(5) 17.956(5) 19.651(5)
alpha, beta, gamma [deg]	110.379(5) 110.782(5) 101.013(5)
V [Ang**3]	4626(2)
Z	1
D(calc) [g/cm**3]	2.203
Mu(MoKa) [/mm]	6.211
F(000)	2942
Crystal Size [mm]	0.15 x 0.20 x 0.25
Data Collection	
Temperature (K)	293
Radiation [Angstrom]	MoKa 0.71073
Theta Min-Max [Deg]	2.0, 28.2

Dataset	-20: 20 ; -23: 23 ; -25: 26
Tot., Uniq. Data, R(int)	50748, 20340, 0.039
Observed Data [I > 2.0 sigma(I)]	13067
Refinement	
Nref, Npar	20340, 1137
R, wR2, S	0.0384, 0.0764, 0.95
w = ^2^(FO^2^)+(0.0298P)^2^] WHERE P=(FO^2^+2FC^2^)/3'	
Max. and Av. Shift/Error	0.02, 0.00
Min. and Max. Resd. Dens. [e/Ang^3]	-0.60, 0.76

Table S5 Computed Structures and Cartesian Coordinates

$[Co_{38}As_{12}(CO)_{50}]^{4-}$ (D_{2h}); the geometry was optimized at the BLYP-D3BJ/SBKJC-ECP level of theory.

Co	1.892512000	1.882369000	1.878680000
As	-1.958318000	-3.175627000	0.000000000
As	3.182192000	0.000000000	1.951644000
As	1.958318000	-3.175627000	0.000000000
As	0.000000000	1.950633000	3.157162000
As	3.182192000	0.000000000	-1.951644000
As	0.000000000	-1.950633000	3.157162000
Co	1.806760000	0.000000000	0.000000000
Co	0.000000000	0.000000000	1.792803000
Co	0.000000000	-1.796418000	0.000000000
Co	1.892512000	-1.882369000	1.878680000
Co	-1.892512000	-1.882369000	1.878680000
Co	1.892512000	-1.882369000	-1.878680000
Co	1.558591000	0.000000000	3.822754000
Co	0.000000000	-3.840341000	1.537866000

Co	0.000000000	-3.840341000	-1.537866000
Co	3.843206000	-1.533590000	0.000000000
Co	5.454969000	0.000000000	-1.349461000
Co	5.454969000	0.000000000	1.349461000
Co	-1.359418000	-5.444648000	0.000000000
Co	0.000000000	1.344380000	5.424736000
Co	0.000000000	-1.344380000	5.424736000
Co	1.359418000	-5.444648000	0.000000000
C	0.000000000	-4.968440000	-2.863578000
O	0.000000000	-5.697407000	-3.828315000
C	-2.318704000	-6.035074000	-1.366086000
O	-3.028089000	-6.458816000	-2.240793000
C	-2.318704000	-6.035074000	1.366086000
O	-3.028089000	-6.458816000	2.240793000
C	0.000000000	-6.825631000	0.000000000
O	0.000000000	-8.057857000	0.000000000
C	0.000000000	-4.968440000	2.863578000

O	0.000000000	-5.697407000	3.828315000
C	2.318704000	-6.035074000	1.366086000
O	3.028089000	-6.458816000	2.240793000
C	-1.370068000	-2.304196000	6.007600000
O	-2.248186000	-3.011054000	6.428397000
C	1.370068000	-2.304196000	6.007600000
O	2.248186000	-3.011054000	6.428397000
C	2.897631000	-2.899240000	2.889941000
O	3.592901000	-3.595867000	3.580176000
C	2.887459000	0.000000000	4.948083000
O	3.855743000	0.000000000	5.673198000
C	1.370068000	2.304196000	6.007600000
O	2.248186000	3.011054000	6.428397000
C	4.963205000	-2.869683000	0.000000000
O	5.682237000	-3.841848000	0.000000000
C	6.037867000	-1.367249000	2.314470000
O	6.455243000	-2.245250000	3.023522000

C	6.037867000	1.367249000	2.314470000
O	6.455243000	2.245250000	3.023522000
C	6.037867000	1.367249000	-2.314470000
O	6.455243000	2.245250000	-3.023522000
C	2.897631000	-2.899240000	-2.889941000
O	3.592901000	-3.595867000	-3.580176000
C	6.037867000	-1.367249000	-2.314470000
O	6.455243000	-2.245250000	-3.023522000
C	2.318704000	-6.035074000	-1.366086000
O	3.028089000	-6.458816000	-2.240793000
C	2.897631000	2.899240000	2.889941000
O	3.592901000	3.595867000	3.580176000
C	-2.897631000	-2.899240000	2.889941000
O	-3.592901000	-3.595867000	3.580176000
C	-1.370068000	2.304196000	6.007600000
O	-2.248186000	3.011054000	6.428397000
Co	-3.843206000	-1.533590000	0.000000000

C	-4.963205000	-2.869683000	0.000000000
O	-5.682237000	-3.841848000	0.000000000
Co	-1.558591000	0.000000000	3.822754000
C	-2.887459000	0.000000000	4.948083000
O	-3.855743000	0.000000000	5.673198000
C	6.853197000	0.000000000	0.000000000
O	8.082858000	0.000000000	0.000000000
C	0.000000000	0.000000000	6.814580000
O	0.000000000	0.000000000	8.046275000
As	1.958318000	3.175627000	0.000000000
As	-3.182192000	0.000000000	-1.951644000
As	-1.958318000	3.175627000	0.000000000
As	0.000000000	-1.950633000	-3.157162000
As	-3.182192000	0.000000000	1.951644000
As	0.000000000	1.950633000	-3.157162000
Co	-1.806760000	0.000000000	0.000000000
Co	0.000000000	0.000000000	-1.792803000

Co	0.000000000	1.796418000	0.000000000
Co	-1.892512000	-1.882369000	-1.878680000
Co	-1.892512000	1.882369000	-1.878680000
Co	1.892512000	1.882369000	-1.878680000
Co	-1.892512000	1.882369000	1.878680000
Co	-1.558591000	0.000000000	-3.822754000
Co	0.000000000	3.840341000	-1.537866000
Co	0.000000000	3.840341000	1.537866000
Co	-3.843206000	1.533590000	0.000000000
Co	-5.454969000	0.000000000	1.349461000
Co	-5.454969000	0.000000000	-1.349461000
Co	1.359418000	5.444648000	0.000000000
Co	0.000000000	-1.344380000	-5.424736000
Co	0.000000000	1.344380000	-5.424736000
Co	-1.359418000	5.444648000	0.000000000
C	0.000000000	4.968440000	2.863578000
O	0.000000000	5.697407000	3.828315000

C	2.318704000	6.035074000	1.366086000
O	3.028089000	6.458816000	2.240793000
C	2.318704000	6.035074000	-1.366086000
O	3.028089000	6.458816000	-2.240793000
C	0.000000000	6.825631000	0.000000000
O	0.000000000	8.057857000	0.000000000
C	0.000000000	4.968440000	-2.863578000
O	0.000000000	5.697407000	-3.828315000
C	-2.318704000	6.035074000	-1.366086000
O	-3.028089000	6.458816000	-2.240793000
C	1.370068000	2.304196000	-6.007600000
O	2.248186000	3.011054000	-6.428397000
C	-1.370068000	2.304196000	-6.007600000
O	-2.248186000	3.011054000	-6.428397000
C	-2.897631000	2.899240000	-2.889941000
O	-3.592901000	3.595867000	-3.580176000
C	-2.887459000	0.000000000	-4.948083000

O	-3.855743000	0.000000000	-5.673198000
C	-1.370068000	-2.304196000	-6.007600000
O	-2.248186000	-3.011054000	-6.428397000
C	-4.963205000	2.869683000	0.000000000
O	-5.682237000	3.841848000	0.000000000
C	-6.037867000	1.367249000	-2.314470000
O	-6.455243000	2.245250000	-3.023522000
C	-6.037867000	-1.367249000	-2.314470000
O	-6.455243000	-2.245250000	-3.023522000
C	-6.037867000	-1.367249000	2.314470000
O	-6.455243000	-2.245250000	3.023522000
C	-2.897631000	2.899240000	2.889941000
O	-3.592901000	3.595867000	3.580176000
C	-6.037867000	1.367249000	2.314470000
O	-6.455243000	2.245250000	3.023522000
C	-2.318704000	6.035074000	1.366086000
O	-3.028089000	6.458816000	2.240793000

C	-2.897631000	-2.899240000	-2.889941000
O	-3.592901000	-3.595867000	-3.580176000
C	2.897631000	2.899240000	-2.889941000
O	3.592901000	3.595867000	-3.580176000
C	1.370068000	-2.304196000	-6.007600000
O	2.248186000	-3.011054000	-6.428397000
Co	3.843206000	1.533590000	0.000000000
C	4.963205000	2.869683000	0.000000000
O	5.682237000	3.841848000	0.000000000
Co	1.558591000	0.000000000	-3.822754000
C	2.887459000	0.000000000	-4.948083000
O	3.855743000	0.000000000	-5.673198000
C	-6.853197000	0.000000000	0.000000000
O	-8.082858000	0.000000000	0.000000000
C	0.000000000	0.000000000	-6.814580000
O	0.000000000	0.000000000	-8.046275000

$[Co_{38}As_{12}(CO)_{50}]^{4-}$ (S_6); the geometry was optimized at the BLYP-D3BJ/SBKJC-ECP level of theory.

As	-2.306708000	-0.201862000	2.978552000
As	2.306708000	0.201862000	-2.978552000
As	2.003089000	-3.117448000	-0.775550000
As	-2.003089000	3.117448000	0.775550000
As	-1.698245000	-3.293450000	0.775550000
As	1.698245000	3.293450000	-0.775550000
As	3.701334000	0.176002000	0.775550000
As	-3.701334000	-0.176002000	-0.775550000
As	-0.978536000	-2.098599000	-2.978552000
As	0.978536000	2.098599000	2.978552000
As	1.328172000	-1.896736000	2.978552000
As	-1.328172000	1.896736000	-2.978552000
Co	0.285440000	-1.436674000	-1.031324000
Co	-0.285440000	1.436674000	1.031324000
Co	1.386916000	-0.471138000	1.031324000
Co	-1.386916000	0.471138000	-1.031324000

Co	-1.101476000	-0.965536000	1.031324000
Co	1.101476000	0.965536000	-1.031324000
Co	2.991982000	-1.021376000	-1.104806000
Co	-2.991982000	1.021376000	1.104806000
Co	0.611453000	-3.101820000	1.104806000
Co	-0.611453000	3.101820000	-1.104806000
Co	0.000000000	0.000000000	3.354936000
Co	0.000000000	0.000000000	-3.354936000
Co	-2.380528000	-2.080444000	-1.104806000
Co	2.380528000	2.080444000	1.104806000
Co	3.267945000	-2.270310000	1.328493000
Co	-3.267945000	2.270310000	-1.328493000
Co	1.568041000	-2.252961000	-3.158819000
Co	-1.568041000	2.252961000	3.158819000
Co	-1.167101000	-2.484444000	3.158819000
Co	1.167101000	2.484444000	-3.158819000
Co	-3.600119000	-1.694968000	1.328493000

Co	3.600119000	1.694968000	-1.328493000
Co	-0.332174000	-3.965278000	-1.328493000
Co	0.332174000	3.965278000	1.328493000
Co	-2.735142000	-0.231483000	-3.158819000
Co	2.735142000	0.231483000	3.158819000
Co	-0.224241000	-4.131295000	-3.979628000
Co	0.224241000	4.131295000	3.979628000
Co	1.968354000	-4.805198000	-2.486226000
Co	-1.968354000	4.805198000	2.486226000
Co	-3.689926000	-1.871449000	3.979628000
Co	3.689926000	1.871449000	-3.979628000
Co	5.145601000	-0.697954000	2.486226000
Co	-5.145601000	0.697954000	-2.486226000
Co	3.465686000	-2.259845000	3.979628000
Co	-3.465686000	2.259845000	-3.979628000
Co	-3.177246000	-4.107244000	2.486226000
Co	3.177246000	4.107244000	-2.486226000

C	-5.311752000	-2.065127000	1.160076000
C	5.311752000	2.065127000	-1.160076000
O	-6.489436000	-2.284583000	0.996944000
O	6.489436000	2.284583000	-0.996944000
C	-5.332112000	-1.164315000	4.027193000
C	5.332112000	1.164315000	-4.027193000
O	-6.435162000	-0.709711000	4.191710000
O	6.435162000	0.709711000	-4.191710000
C	-3.302243000	-1.670871000	5.717703000
C	3.302243000	1.670871000	-5.717703000
O	-3.127894000	-1.468677000	6.890871000
O	3.127894000	1.468677000	-6.890871000
C	-4.241872000	-3.738253000	4.068833000
C	4.241872000	3.738253000	-4.068833000
O	4.961554000	4.408483000	-4.811543000
O	-4.961554000	-4.408483000	4.811543000
C	-0.911221000	-3.346616000	4.675152000

C	0.911221000	3.346616000	-4.675152000
O	-0.697898000	-3.910193000	5.723241000
O	0.697898000	3.910193000	-5.723241000
C	-2.344279000	-5.545210000	3.142290000
C	2.344279000	5.545210000	-3.142290000
O	-1.882485000	-6.577155000	3.556550000
O	1.882485000	6.577155000	-3.556550000
C	3.098138000	-2.024391000	5.717703000
C	-3.098138000	2.024391000	-5.717703000
O	-2.835858000	1.974497000	-6.890871000
O	2.835858000	-1.974497000	6.890871000
C	3.674382000	-4.035587000	4.027193000
C	-3.674382000	4.035587000	-4.027193000
O	3.832209000	-5.218158000	4.191710000
O	-3.832209000	5.218158000	-4.191710000
C	0.947234000	-4.726827000	1.692069000
C	-0.947234000	4.726827000	-1.692069000

O	1.177347000	-5.838986000	2.088262000
O	-1.177347000	5.838986000	-2.088262000
C	4.444328000	-3.567548000	1.160076000
C	-4.444328000	3.567548000	-1.160076000
O	5.223224000	-4.477725000	0.996944000
O	-5.223224000	4.477725000	-0.996944000
C	6.521754000	-1.357309000	1.546627000
C	-6.521754000	1.357309000	-1.546627000
O	7.510648000	-1.707729000	0.955845000
O	-7.510648000	1.707729000	-0.955845000
C	-0.867424000	-5.632675000	-1.160076000
C	0.867424000	5.632675000	1.160076000
O	-1.266211000	-6.762307000	-0.996944000
O	1.266211000	6.762307000	0.996944000
C	2.085412000	-6.326659000	-1.546627000
C	-2.085412000	6.326659000	1.546627000
O	2.276387000	-7.358277000	-0.955845000

O	-2.276387000	7.358277000	0.955845000
C	3.630153000	-4.802810000	-3.142290000
C	-3.630153000	4.802810000	3.142290000
O	4.754741000	-4.918857000	-3.556550000
O	-4.754741000	4.918857000	3.556550000
C	-0.204105000	-3.695262000	-5.717703000
C	0.204105000	3.695262000	5.717703000
O	-0.292035000	-3.443174000	-6.890871000
O	0.292035000	3.443174000	6.890871000
C	-3.619935000	-3.183742000	-1.692069000
C	3.619935000	3.183742000	1.692069000
O	-4.468037000	-3.939105000	-2.088262000
O	4.468037000	3.939105000	2.088262000
C	-1.657730000	-5.199902000	-4.027193000
C	1.657730000	5.199902000	4.027193000
O	-2.602953000	-5.927869000	-4.191710000
O	2.602953000	5.927869000	4.191710000

C	-4.436341000	-4.969350000	1.546627000
C	4.436341000	4.969350000	-1.546627000
O	5.234261000	5.650547000	-0.955845000
O	-5.234261000	-5.650547000	0.955845000
C	4.567169000	-1.543085000	-1.692069000
C	-4.567169000	1.543085000	1.692069000
O	5.645384000	-1.899880000	-2.088262000
O	-5.645384000	1.899880000	2.088262000
C	0.000000000	0.000000000	5.114373000
C	0.000000000	0.000000000	-5.114373000
O	0.000000000	0.000000000	-6.316686000
O	0.000000000	0.000000000	6.316686000
C	2.442644000	-2.462449000	-4.675152000
C	-2.442644000	2.462449000	4.675152000
O	3.037377000	-2.559494000	-5.723241000
O	-3.037377000	2.559494000	5.723241000
C	-3.353865000	-0.884167000	-4.675152000

C	3.353865000	0.884167000	4.675152000
O	-3.735276000	-1.350699000	-5.723241000
O	3.735276000	1.350699000	5.723241000
C	1.116486000	-5.542696000	-4.068833000
C	-1.116486000	5.542696000	4.068833000
O	1.337082000	-6.501073000	-4.811543000
O	-1.337082000	6.501073000	4.811543000
C	-5.358359000	1.804442000	-4.068833000
C	5.358359000	-1.804442000	4.068833000
O	-6.298636000	2.092590000	-4.811543000
O	6.298636000	-2.092590000	4.811543000
C	5.974432000	0.742400000	3.142290000
C	-5.974432000	-0.742400000	-3.142290000
O	-6.637226000	-1.658298000	-3.556550000
O	6.637226000	1.658298000	3.556550000
